# Chronic Pain and Regional Anesthesia: A Call to Action!

**DOI:** 10.3390/jcm12051955

**Published:** 2023-03-01

**Authors:** Alessandro De Cassai, Federico Geraldini

**Affiliations:** Anesthesia and Intensive Care Unit, University-Hospital of Padua,13, Gallucci Street, 35121 Padua, Italy

Chronic pain is an unwanted companion in the lives of millions of people worldwide, and findings show that more than one in fiveadults in America experience chronic pain [1]. Chronic pain is characterized by persistent or recurrent pain that lasts for more than three months and can significantly impair a person’s quality of life and increase the number of days lostfrom work, being a burden for both individuals and society. Chronic pain can stemfrom various causes, including trauma, surgery, inflammation, nerve damage, and cancer. Despite the availability of several treatment options, chronic pain management remains a significant healthcare challenge. Regional anesthesia is a promising approach to managing chronic pain, and recent research has shown its potential benefits [2]. In this Editorial, we will discuss the role of regional anesthesia in chronic pain management, current knowledge, and possible future research. Regional anesthesia involves the injection of local anesthetics or other drugs around specific nerves or nerve plexuses to block pain transmission to the brain. This technique provides targeted pain relief and has been used successfully in acute pain management, such as in postoperative pain. However, its use in chronic pain management is less wellestablished. Recent studies have shown that regional anesthesia can provide effective and long-lasting pain relief for various chronic pain conditions, including neuropathic pain, complex regional pain syndrome, and cancer-related pain [3]. One of the advantages of regional anesthesia in chronic pain management is its ability to provide targeted pain relief. Unlike systemic medications which can affect the entire body, regional anesthesia targets the specific nerves responsible for the pain [4]. This approach reduces the risk of adverse effects, such as sedation, respiratory depression, and gastrointestinal problems, commonly associated with systemic medications. Regional anesthesia also provides more prolonged pain relief than systemic medications, with a lower risk of tolerance and addiction. In recent years, several studies have investigated the use of regional anesthesia in chronic pain management. For example, a randomized controlled trial published in Anesthesiology in 2018 compared the efficacy of epidural steroid injection (ESI) with ultrasound-guided erector spinae plane block (ESPB) in managing chronic lumbar radicular pain. The study found that both techniques provided significant pain relief, but the ESPB group reported better pain control and functional improvement [5]. Similarly, a systematic review and meta-analysis published in Regional Anesthesia and Pain Medicine in 2021 found that peripheral nerve blocks were effective in managing chronic shoulder pain, with a low risk of adverse effects [6]. Another area where regional anesthesia has shown promise is cancer-related pain management. Cancer-related pain is a complex and challenging condition that can significantly affect a person’s quality of life. The use of systemic opioids is the standard approach to managing cancer-related pain, but it is associated with several adverse effects, including sedation, respiratory depression, and constipation. Regional anesthesia can provide effective pain relief while reducing the need for systemic opioids, with a lower risk of adverse effects. For example, a randomized controlled trial published in Pain Medicine in 2020 investigated the efficacy of paravertebral block (PVB) in managing cancer-related pain in patients with advanced lung cancer. The study found that PVB provided significant pain relief and improved functional status, with a lower opioid consumption [7].

While regional anesthesia has shown promise in chronic pain management, there are still several challenges that need to be addressed. One of the challenges is the lack of standardization in the technique and dosing of regional anesthesia. Regional anesthesia techniques vary depending on the type and location of pain, and there is currently no consensus on the optimal technique or dosing. This variability can affect the efficacy and safety of the technique and may limit its widespread adoption. Future research should focus on standardizing regional anesthesia techniques and dosing to ensure consistent and optimal pain relief.

Another challenge is the limited availability of trained personnel to perform regional anesthesia. Regional anesthesia requires specialized training and expertise, and many healthcare providers may not have the necessary skills to perform the technique and this limitation can affect the accessibility of regional anesthesia even if complication rate, especially for some techniques, is low [8].

It is clear and of paramount importance that research is still needed in this interesting area, and in the present Special Issue of the Journal of Clinical Medicine we hope to collect several and variouspathways for present and future research.

One area of future research is focused on identifying the optimal dose and duration of local anesthetics used in regional anesthesia. While there is some evidence to suggest that higher doses of local anesthetics may provide better pain relief, there are also concerns about the potential for toxicity and nerve damage. Finding the right balance between efficacy and safety will be an important consideration in the development of future studies.

Another area of research is focused on identifying the patient characteristics that predict responsiveness to regional anesthesia. Some studies have suggested that patients with certain pain conditions, such as neuropathic pain or complex regional pain syndrome, may be more responsive to regional anesthesia than others. Understanding these predictors could help clinicians to identify the patients most likely to benefit from this technique.

In addition to these areas of research, there is also growing interest in combining regional anesthesia with other treatment modalities to optimize pain relief. For example, one recent study published in Pain Medicine evaluated the use of ultrasound-guided transversus abdominis plane block in combination with pharmacotherapy for the management of chronic abdominal pain. The study found that this combination approach was more effective in reducing pain intensity and improving quality of life than pharmacotherapy alone.

There are also ongoing efforts to develop new regional anesthesia techniques that can provide targeted pain relief to specific areas of the body. One example is the use of ultrasound-guided pulsed radiofrequency ablation, which uses high-frequency electrical currents to selectively disrupt the function of pain-transmitting nerves. While this technique is still in the early stages of development, initial studies have shown promising results in the management of chronic pain.

Finally, there is a need for more research on the long-term effects of regional anesthesia on chronic pain. While many studies have evaluated the short-term efficacy of these techniques, there is still much to be learned about their long-term effects on pain intensity, quality of life, and functional status. Understanding these effects will be critical for developing more effective and sustainable chronic pain management strategies.

In conclusion, regional anesthesia represents a valuable tool in the management of chronic pain. As research in this area continues to expand, there are several future studies that could enhance our understanding of this technique’s role in pain management. By identifying the optimal dosing and patient characteristics that predict responsiveness to regional anesthesia, combining regional anesthesia with other treatment modalities, developing new techniques, and evaluating the long-term effects of regional anesthesia, we can continue to improve the quality of life of those living with chronic pain.
